# DNA Vaccine Expressing Conserved Influenza Virus Proteins Protective Against H5N1 Challenge Infection in Mice

**DOI:** 10.3201/eid0808.010476

**Published:** 2002-08

**Authors:** Suzanne L. Epstein, Terrence M. Tumpey, Julia A. Misplon, Chia-Yun Lo, Lynn A. Cooper, Kanta Subbarao, Mary Renshaw, Suryaprakash Sambhara, Jacqueline M. Katz

**Affiliations:** * Food and Drug Administration Rockville, Maryland, USA; † United States Department of Agriculture, Athens, Georgia, USA; ‡ Centers for Disease Control and Prevention, Atlanta, Georgia, USA

**Keywords:** influenza, influenza vaccine, DNA vaccines, viral antibodies, immunity, cellular, vaccination, RNA virus infections, influenza A virus, avian influenza A virus, mice

## Abstract

Influenza vaccination practice, which is based on neutralizing antibodies, requires being able to predict which viral strains will be circulating. If an unexpected strain, as in the 1997 H5N1 Hong Kong outbreak, or even a pandemic emerges, appropriate vaccines may take too long to prepare. Therefore, strategies based on conserved influenza antigens should be explored. We studied DNA vaccination in mice with plasmids expressing conserved nucleoprotein (NP) and matrix (M) from an H1N1 virus. After vaccination, mice were challenged with A/H5N1 viruses of low, intermediate, and high lethality. A/NP+A/M DNA vaccination reduced replication of A/Hong Kong/486/97 (HK/486), a nonlethal H5N1 strain, and protected against lethal challenge with more virulent A/Hong Kong/156/97 (HK/156). After HK/156 exposure, mice survived rechallenge with A/Hong Kong/483/97 (HK/483), although the DNA vaccination alone protected poorly against this highly virulent strain. In the absence of antigenically matched hemagglutinin-based vaccines, DNA vaccination with conserved influenza genes may provide a useful first line of defense against a rapidly spreading pandemic virus.

The 1997 outbreak of H5N1 avian influenza in humans in Hong Kong (1,2) caused alarm because it involved highly pathogenic strains of an influenza subtype to which humans lack immunity. This outbreak led to fears about inability to control a pandemic if a new strain should spread efficiently from human to human. Although prevention by vaccination is more desirable than treatment after infection, conventional immunization strategies have major limitations.

Neutralizing antibodies are specific to subtype and often strain, so vaccination based on eliciting such antibodies requires accurate prediction of the viral strains that will circulate during the influenza season and leaves little time for vaccine preparation. Even with usual epidemic strains, difficulties and delays in the production of an adequate vaccine supply have occurred in some years (3). A rapidly developing pandemic would shorten the timeframe to identify the viral strain and prepare an antigenically matched vaccine, while the need to vaccinate an entirely naïve population would exacerbate vaccine production and supply issues. In addition, H5 vaccine candidates, either H5 recombinant protein or a conventional surface antigen vaccine prepared from apathogenic H5N3 virus, have shown suboptimal immunogenicity in human trials (4,5).

A recent report on the molecular basis for virulence of H5N1 viruses (6) was accompanied by an article that discussed related public health issues, in which Laver and Garman (7) addressed the problem of how to control pandemics and concluded that currently “the most promising first line of defense” is use of antiviral drugs. These drugs, however, reduce symptoms and duration of disease only partially (8), and their effectiveness during H5N1 infection is unknown. Laver and Garman further commented that various experimental vaccines, including DNA vaccines, may be more promising for pandemic control. These statements highlight the fact that additional approaches are needed to produce effective vaccines for H5N1 or other new subtypes (9).

Vaccines using conserved components of influenza A virus can induce protection against many influenza A strains, including those of divergent subtypes. Animal studies have demonstrated potent and long-lasting heterosubtypic immunity, that is, exposure to a virus of one subtype protects against challenge infection with another subtype (10–15). The mechanisms of heterosubtypic immunity are not completely understood but likely include both T-cell immunity, in particular CD8+ cytotoxic T-lymphocytes (CTL) (16,17) and CD4+ T cells (13), as well as antibodies to conserved epitopes (18). Heterosubtypic immunity has been reported in humans (19, 20), but its effectiveness and duration are unknown. Animal studies may show ways to optimize induction of heterosubtypic immunity, which could then be tested in humans. Heterosubtypic immunity induced by virus can protect against H5N1 infection in animals (21), and human T cells specific for antigens of an H1N1 virus, including nucleoprotein (NP) and matrix (M), can lyse target cells infected with H5N1 virus (22). In addition, exposure to H9N2 virus can induce heterosubtypic protection against H5N1 challenge in chickens (23) and mice (24).

 DNA vaccination can target immune responses to epitopes that are highly conserved in influenza A viruses, while avoiding the risks of live-virus vaccines. We and others have previously shown that DNA constructs expressing conserved influenza proteins induce antibody and T-cell responses and protect against H3N2 heterosubtypic challenge (25–27). Both CD4+ and CD8+ T cells play roles in this protective immunity. DNA vaccination has also been studied in the H5N1 system, although largely with constructs expressing HA. DNA constructs expressing H5 HA can protect against lethal H5N1 challenge in mice (28). In lethal challenge experiments with chickens, an H5 HA construct protected fully and a construct expressing NP of an H5N8 virus protected partially (29,30). However, DNA vaccines expressing heterosubtypic antigens have not been studied in the H5N1 system.

Studies of challenge with H5N1 viruses from the 1997 Hong Kong outbreak must take into account their phenotypic diversity. While all these viruses were highly pathogenic in chickens, two main pathogenicity phenotypes were observed in mice (31,32). Viruses of the two types were studied for histopathology, viral titers in various tissues, and lethality in mice. The H3N2 viruses A/Udorn or X31 were used for comparison in some cases. Some isolates, represented by A/Hong Kong/483/97 (HK/483), were lethal even at modest doses, replicating in multiple organs, including the brain, liver, spleen, and kidney, after intranasal administration (31), resulting in pathology of respiratory tissue and the heart, and producing immune effects (33). Other isolates, represented by A/Hong Kong/486/97 (HK/486), replicated only in the respiratory tract and were not lethal. One virus, A/Hong Kong/156/97 (HK/156), did not fit readily into either group, requiring higher doses to infect or kill mice in one of the studies (31) and showing some spread to nonrespiratory sites but more limited spread than was seen with HK/483 (31,32). HK/483 and HK/156, but not HK/486, were isolated from lethal infections in the original human cases.

In this study, we extended DNA vaccination based on conserved influenza components to heterosubtypic challenge with H5N1 virus. We investigated whether the broadly cross-reactive immunity induced by immunization of mice with DNA expressing NP and M from a mouse-adapted human H1N1 virus, A/Puerto Rico/8/34 (A/PR/8), could control infection with a range of H5N1 viruses.

## Materials and Methods

Plasmid VR1012 was obtained from Vical Inc. (San Diego, CA) under a Materials Transfer Agreement. Full-length influenza genes for NP and M of A/PR/8 were prepared and inserted into VR1012 as described previously (27). The plasmid B/NP expresses the full-length NP gene from B/Ann Arbor/1/86 (B/AA), derived from a baculovirus vector generated by Rota et al. (34) and subcloned into VR1012. Plasmid DNA was prepared and tested as described (27). Endotoxin levels were <1 EU/100 μg dose.

H5N1 viruses used in this study were HK/156, HK/483, HK/485, and HK/486 (31). Other viruses used were H1N1 virus A/PR/8; reassortant virus X-31 with surface glycoproteins of A/Aichi/2/68 (H3N2) and internal proteins of A/PR/8 virus; and B/AA. The A/PR/8 and X-31 stocks were mouse adapted by passage through mouse lungs. Virus stocks were propagated in the allantoic cavity of embryonated hen eggs at 37°C for 24 hr (H5N1 viruses) or 34°C for 48–72 hr (other viruses). Fifty-percent egg infectious dose (EID_50_) titers and mouse infectious dose (MID_50_) titers were determined by serial titration in eggs or mouse lungs, respectively, and calculated by the method of Reed and Muench (35). All experiments with infectious H5N1 viruses were conducted under BSL-3+ containment, including work in animals.

BALB/c female mice were purchased from the Division of Cancer Treatment, National Cancer Institute, Frederick, Maryland, or from Jackson Laboratories, Bar Harbor, Maine. DNA was injected intramuscularly, 100 µg/mouse of each plasmid, three times at 2-week intervals, starting at 6–7 weeks of age. Approximately 1 week after the last immunization, mice were shipped from the Food and Drug Administration to Centers for Disease Control and Prevention or U.S. Department of Agriculture, allowed to rest for approximately a week, challenged under containment conditions with CO_2_ anesthesia, and monitored for weight loss and death. For viral titers, lung and brain tissues were collected 6 days postchallenge and frozen.

Enzyme-linked immunosorbent assay (ELISA) was performed as described previously (15) on plates coated with lysates of influenza virus–infected cells. Hemagglutination inhibition (HI) was performed by standard methods with sera pretreated with receptor-destroying enzyme (36).

Thawed tissues were homogenized in 1 mL of sterile phosphate-buffered saline. Clarified lung, brain, kidney, and nose homogenates were titrated for virus infectivity in 10-day-old embryonated eggs (EID_50_) from initial dilutions of 1:10 (lungs and nose) or 1:2 (brain and kidney), with positive eggs identified by hemagglutination. Detection limits were 10^1.2^ EID_50_/mL for lung and nose, and 10^0.8^ EID_50_/mL for brain and kidney. Enzyme-linked immunospot assay (ELISPOT) for interferon-γ (IFN-γ) secreting cells were performed as described previously (37).

For CTL assays, splenocytes were restimulated in vitro and target cells prepared as described (38). CTL activity was measured by lactate dehydrogenase (LDH) release (CytoTox 96 Non-Radioactive Cytotoxicity Assay kit G170, Promega Corp., Madison, WI). Results were calculated as:

% Lysis = Experimental-Effector Spontaneous-Target Spontaneous X 100

**Figure 1:**
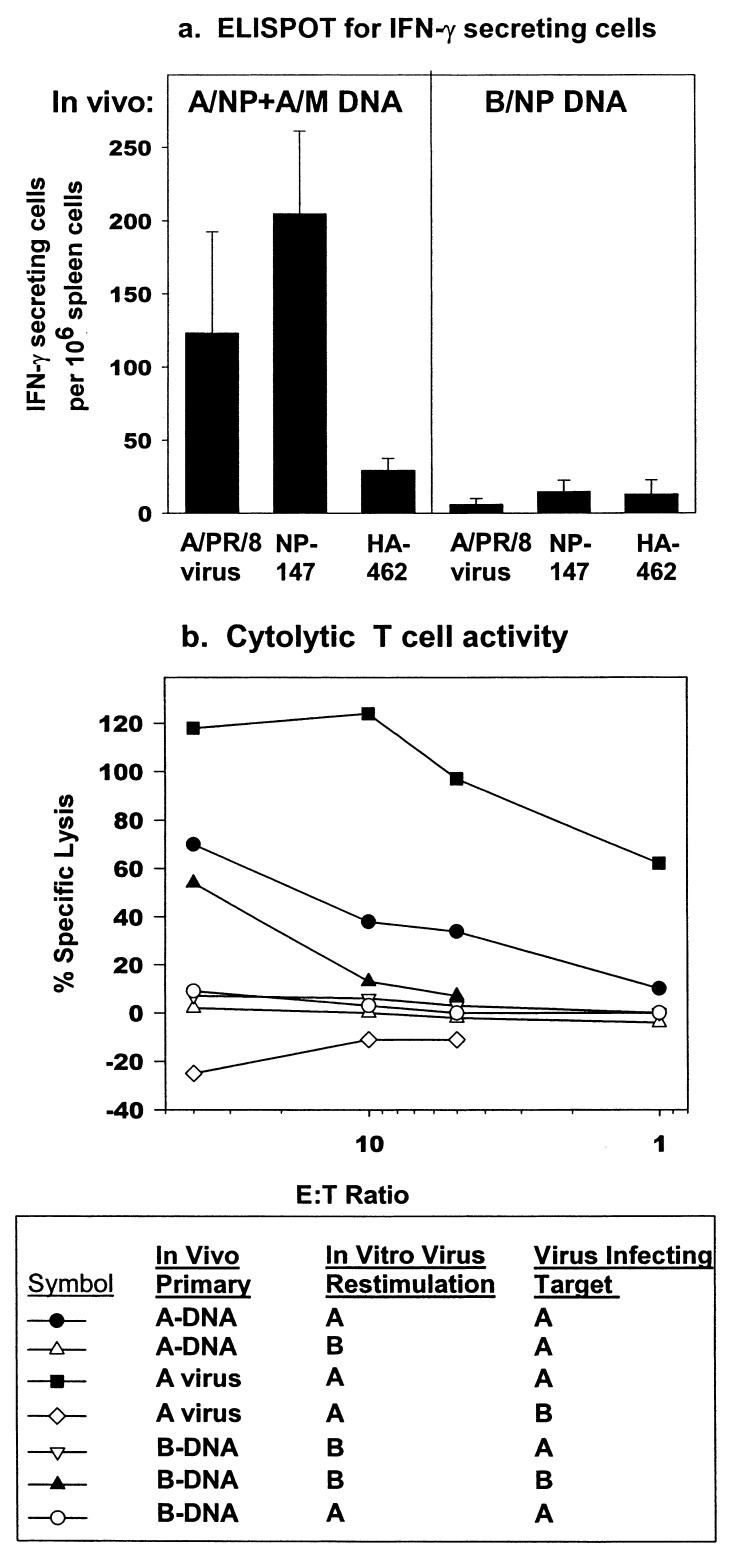
DNA vaccination induces T cell responses. a) Enzyme-linked immuno spot (ELISPOT) assay for interferon-γ (IFN-γ) secreting cells. Mice were immunized three times with A/NP+A/M or influenza B nucleoprotein DNA (B/NP DNA) intramuscularly. Spleen cells were analyzed by ELISPOT, using peptides at 1 μg/ml or A/PR/8 live virus. Results are the mean of three experiments. No response to A/PR/8 virus occurred in one experiment. Concanavalin A (Con A) responses: A/NP+A/M groups, >274 for all experiments; B/NP groups, >329 for all experiments. b) Cytotoxic T cell assay. Mice were vaccinated as above or with live A/PR/8 virus given on the day of the second DNA injection. Spleens were harvested 2½ weeks after the third DNA injection. Spleen cells were restimulated in vitro with live A/PR/8 or B/AA. After 7 days of culture, restimulated effector cells at various ratios were mixed with P815 target cells infected with A/PR/8 or B/AA, and lactate dehydrogenase (LDH) release measured.

Target Maximum-Target Spontaneous

where target maximum represents target cells plus Promega lysis solution containing detergent. Maximum cytotoxicity occasionally exceeds 100% (Figure 1). The addition of targets may alter spontaneous release from effectors, or detergent lysis may differ from CTL-mediated lysis, but relative CTL activity was consistent.

## Results

Mice were immunized with a mixture of plasmids encoding A/NP+A/M, intended to provide greater protection than a single antigen (27,39). Plasmid DNA without an insert is often used as a control; although we used it initially, we later prepared a construct expressing the NP gene of influenza B/AA as a specificity control. The B/AA virus is only distantly related antigenically to influenza A, and no cross-protection is seen between influenza A and B viruses. The control plasmid expressing B/NP protects against challenge with influenza B, as shown by reduction in lung viral titers (40).

A/NP+A/M DNA induced antibodies against homologous A/PR/8 proteins (geometric mean ELISA titer 761), with no cross-reactivity to influenza B proteins (all titers <20). Mice immunized with B/NP DNA had comparable titers of antibody to influenza B proteins, with no cross-reactivity to A/PR/8 proteins.

DNA immunization activated T cells in an antigen-specific manner by two measures, ELISPOT of IFN-γ secreting cells and CTL activity. Splenocytes from mice immunized with A/NP+A/M DNA generated an IFN-γ ELISPOT response when restimulated with NP_147_ peptide (the dominant CTL epitope in BALB/c mice), A/PR/8 virus, or concanavalin A (Con A), but not with control HA_462_ peptide, demonstrating antigen specificity (Figure 1a). Mice immunized with B/NP DNA did not respond to restimulation with either peptide or with A/PR/8 but did respond to Con A, indicating the cells were functional.

Antigen-specific CTL responses to DNA immunization were seen after in vitro restimulation (Figure 1b). Cells from mice immunized with A/NP+A/M DNA lysed A/PR/8-infected targets if they had been restimulated with A/PR/8 but not with B/AA. Controls immunized with B/NP DNA and restimulated with B/AA generated cytolytic activity detectable on influenza B-infected targets but not A/PR/8-infected targets.

A/NP+A/M DNA immunization was tested for protection against an H5N1 challenge virus of low virulence, HK/486. HK/486 is not lethal for mice, so control of virus replication was measured. A/NP+A/M vaccination reduced replication of HK/486 virus in the lungs approximately 17-fold, compared with viral titers in mice vaccinated with control DNA or unimmunized mice (Table, highly significant by Analysis of variance (ANOVA); see legend). As expected, infection of mice with X-31 virus induced substantial heterosubtypic immunity, reducing lung virus titers by approximately 3,000-fold compared with unvaccinated controls.

**Table:** Effect of DNA vaccination on replication of HK/486 challenge virus in mouse lungs^a^

Immunization	No. mice	Lung titer +/- SE
Expt 1A		
A/NP+A/M DNA	6	5.7 ± 0.33
B/NP + blank DNA	6	6.9 ± 0.18^b^
None	6	6.9 ± 0.22^c^
Expt 1B		
Live X-31 virus	4	3.6 ± 0.36^d^
None	4	7.1 ± 0.1

^a^Mice were immunized intramuscularly with 100 μg each of influenza A nucleoprotein and matrix DNA (A/NP+A/M DNA) or controls with 100 μg each of influenza B nucleoprotein DNA (B/NP)+blank DNA (total dose 200 μg/mouse on each occasion), three times at 2-week intervals. Two weeks after the last dose of DNA, mice were challenged with 100 mouse infectious dose (MID)_50_ of HK/486 intranasally. X31 virus-primed mice and their controls were challenged along with DNA-vaccinated mice. On day 6 after challenge, mice were sacrificed and lungs collected for titration of virus infectivity. ^b^Differs significantly from A/NP+A/M group by analysis of variation (ANOVA), p=0.0082. ^c^Differs significantly from A/NP+A/M group by ANOVA, p=0.011. ^d^Differs significantly from unimmunized group by ANOVA, p<0.001.

**Figure 2:**
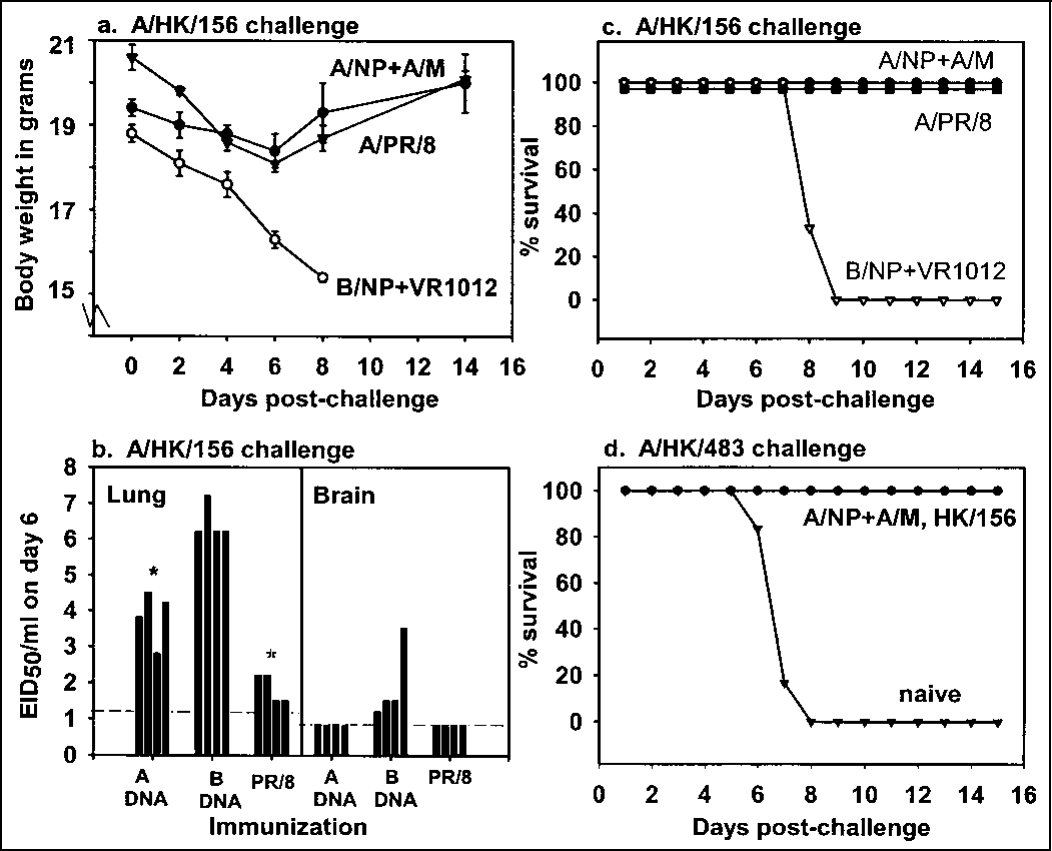
Mice immunized with influenza A nucleoprotein and matrix DNA (A/NP+A/M DNA) are protected against lethal A/Hong Kong/156/97 (HK/156) challenge. Mice were vaccinated as in Figure 1 with A/NP+A/M DNA, with influenza B nucleoprotein DNA (B/NP+blank DNA), or with 100 mouse infectious dose (MID)_50_ of influenza A/Puerto Rico8/34 (A/PR/8) live virus. Sixteen days after the last dose of DNA, mice were challenged with 10,000 MID_50_ of HK/156/97 intranasally. a) Monitoring of morbidity by body weight loss. b) Viral titers of lung and brain homogenates. Each bar represents the result for one mouse. Dashed lines indicate detection limits. Compared to the B/NP DNA controls, lung titers were significantly reduced in the A/NP+A/M DNA group (p=0.001, analysis of variation (ANOVA)) and the A/PR/8 group (p<0.001, ANOVA). c) Survival after challenge with HK/156. d) Survival after rechallenge with 100 MID_50_ of HK/483 of mice primed with A/NP+A/M DNA and which had all survived the previous HK/156 infection.

 Next, we tested the ability of A/NP+A/M DNA vaccination to protect against HK/156, an H5N1 challenge virus of intermediate virulence. Mice vaccinated with A/NP+A/M DNA had only minor weight loss after challenge, while mice vaccinated with control DNA lost weight dramatically (Figure 2a). Four of mice per group were euthanized at day 6 after challenge to measure virus replication in lungs and brains. A/NP+A/M DNA immunization reduced lung titers by over two logs (approximately 500-fold, highly significant by ANOVA, Figure 2b). As expected (21), immunization with A/PR/8 virus also reduced lung titers substantially. Reductions in brain titers were not statistically significant because virus titers in the brain were low even in B/NP DNA-immunized controls.

Mice vaccinated (six per group) with A/NP+A/M DNA all survived a HK/156 challenge dose lethal to controls, as did A/PR/8-primed mice (Figure 2c). Thus, DNA vaccination with conserved components is effective not only against strains of low virulence like HK/486 but also against a lethal strain. However, A/NP+A/M DNA vaccination was not protective against challenge with 100 MID_50_ of highly virulent A/HK/483 (none of six mice survived). An additional experiment used 100 MID_50_ and a lower challenge dose of HK/483 to determine whether A/NP+A/M DNA vaccination could protect against a less extreme challenge. Challenge with 100 MID_50_ of HK/483 again killed all the mice vaccinated with A/NP+A/M DNA (0/8 survived). With a challenge dose of 10 MID_50_, four of eight mice vaccinated with A/NP+A/M DNA survived, but zero of eight given B/NP DNA and zero of eight naïve controls survived. These results suggest some protective effect, though the numbers are not statistically significant. Preliminary testing of viral titers in lung, nose, kidney, and brain at day 6 showed significant differences in the lungs and noses between A/NP+A/M immunized mice and controls, suggesting some impact of the immunization (data not shown).

A/NP+A/M DNA-vaccinated mice that survived HK/156 infection (above) were rechallenged 14 weeks later with 100 MID_50_ of the virulent HK/483 strain. Since mice vaccinated with control DNA had all died after HK/156 challenge, a group of naïve animals was added to the HK/483 challenge to confirm lethality of the challenge dose. All mice primed with A/NP+A/M DNA and subsequently exposed to HK/156 survived this HK/483 challenge, whereas all naïve mice died by day 8 (Figure 2d).

Anti-HA (H5) antibodies induced by HK/156 exposure might account for the protection against HK/483 infection. To assess this possibility, we tested for HI reactivity in sera from the mice after HK/156 exposure but before HK/483 challenge. All mice immunized with A/NP+A/M DNA and then exposed to HK/156 had antibodies reactive with HK/156 and cross-reactive with HK/483 and HK/485 viruses in HI, while control naïve mice had no detectable antibody (data not shown).

## Discussion

The most straightforward approach to vaccination against a newly emerging influenza subtype is use of inactivated virus or recombinant HA. However, if antigenically matched vaccines were not available in time or in sufficient quantity, other options would be important. Our study examines one of these.

DNA vaccination using genes for conserved antigens could have several advantages. The constructs could be available at any time. Plasmid production in bacteria is more consistent than growth of different viruses in eggs, and a cold chain might not be necessary for storage. To explore the usefulness of this approach, we studied the ability of NP+M DNA vaccines derived from A/PR/8 (H1N1) to protect against H5N1 challenge.

Vaccination with A/NP+A/M DNA readily induced antigen-specific antibody and T-cell responses, as shown previously (26,27). We investigated the potential for A/NP+A/M DNA vaccination to control infection by H5N1 viruses of modest (HK/486), intermediate (HK/156), and very high (HK/483) virulence phenotypes. Upon challenge with HK/486, a strain that is not lethal in mice, lung titers were reduced approximately 17-fold. In previous work, even a 5- to 10-fold reduction in peak lung virus titers correlated with immunity protective against lethal challenge (14), so a 17-fold reduction and the accompanying difference in kinetics of viral clearance could alter biologic outcomes. In a test of its effectiveness, the vaccination provided benefit in the case of lethal challenge with HK/156, resulting in 100% survival and minimal morbidity as measured by weight loss, while unvaccinated controls demonstrated large weight losses and 100% death rates. After surviving HK/156 infection, the mice were resistant to lethal HK/483 challenge. Antibodies to HK/156 were demonstrated by HI to be present and cross-reactive with HK/483 virus before HK/483 challenge, which might account for the protection against HK/483. Of mice vaccinated only with A/NP+A/M DNA, half survived challenge with a dose of HK/483 lethal to all controls. While not statistically significant, the trend suggests some impact from immunization.

Regarding immune mechanisms of protection by A/NP+A/M DNA vaccination, candidates include CTL specific for NP (17) and antibodies to the N-terminal portion of M2 (18). Containing an infection with the kinetics of HK/483 may be difficult because it reaches near peak titers in as little as 24 hours. Only neutralizing antibody may be effective that early. Antigen presentation and reactivation of T-cell effectors take several days. However, when T cells specific for viral antigens are expanded substantially, they can reduce replication of highly lethal influenza viruses and clear infection more rapidly (41).-

Comparing amino acid sequences in GenBank from viruses of five subtypes, NPs were >90% identical, with considerable conservation of known dominant CTL epitopes. M1 sequences were >94% conserved, while M2 sequences varied somewhat more. However, not all protective epitopes are known, and even single mutations can alter protective epitopes. Therefore, studies like the present one are necessary for establishing the range of virus strains against which a vaccine can work.

H5 viruses differ in virulence, and one cannot predict which strain might emerge in a future pandemic. With the threat of a pandemic and suboptimal existing vaccine candidates, new approaches to influenza vaccination should be considered. Our results suggest that DNA vaccination with conserved components has the potential to ameliorate disease caused by H5N1 viruses. The immunity induced by this mode of DNA vaccination does not completely prevent infection but passed the stringent test of protecting against lethal H5N1 challenge. Vaccines inducing neutralizing antibody could be administered subsequently to confer immunity against even the most virulent strains. In the absence of an antigenically matched HA-based vaccine, this approach might be useful as a first line of defense against a rapidly spreading influenza pandemic and should be further explored.
